# Assessing the Contribution of an HtrA Family Serine Protease During *Borrelia turicatae* Mammalian Infection

**DOI:** 10.3389/fcimb.2019.00290

**Published:** 2019-08-13

**Authors:** Clay D. Jackson-Litteken, Amanda K. Zalud, C. Tyler Ratliff, Jacob I. Latham, Travis J. Bourret, Job E. Lopez, Jon S. Blevins

**Affiliations:** ^1^Department of Microbiology and Immunology, University of Arkansas for Medical Sciences, Little Rock, AR, United States; ^2^Department of Medical Microbiology and Immunology, Creighton University, Omaha, NE, United States; ^3^Section of Tropical Medicine, Department of Pediatrics, Baylor College of Medicine and Texas Children's Hospital, Houston, TX, United States

**Keywords:** *Borrelia*, relapsing fever, relapsing fever borrelia, BtpA, HtrA, BhpA, oxidative stress

## Abstract

Tick-borne relapsing fever (TBRF), characterized by recurring febrile episodes, is globally distributed and among the most common bacterial infections in some African countries. Despite the public health concern that this disease represents, little is known regarding the virulence determinants required by TBRF *Borrelia* during infection. Because the chromosomes of TBRF *Borrelia* show extensive colinearity with those of Lyme disease (LD) *Borrelia*, the exceptions represent unique genes encoding proteins that are potentially essential to the disparate enzootic cycles of these two groups of spirochetes. One such exception is a gene encoding an HtrA family protease, BtpA, that is present in TBRF *Borrelia*, but not in LD spirochetes. Previous work suggested that *btpA* orthologs may be important for resistance to stresses faced during mammalian infection. Herein, proteomic analyses of the TBRF spirochete, *Borrelia turicatae*, demonstrated that BtpA, as well as proteins encoded by adjacent genes in the *B. turicatae* genome, were produced in response to culture at mammalian body temperature, suggesting a role in mammalian infection. Further, transcriptional analyses revealed that *btpA* was expressed with the genes immediately upstream and downstream as part of an operon. To directly assess if *btpA* is involved in resistance to environmental stresses, *btpA* deletion mutants were generated. *btpA* mutants demonstrated no growth defect in response to heat shock, but were more sensitive to oxidative stress produced by *t-*butyl peroxide compared to wild-type *B. turicatae*. Finally, *btpA* mutants were fully infectious in a murine relapsing fever (RF) infection model. These results indicate that BtpA is either not required for mammalian infection, or that compensatory mechanisms exist in TBRF spirochetes to combat environmental stresses encountered during mammalian infection in the absence of BtpA.

## Introduction

Relapsing fever (RF), caused by spirochetes belonging to the genus *Borrelia*, is characterized by recurrent febrile episodes accompanied by non-specific symptoms including headache, nausea, vomiting, and diarrhea (Ross and Milne, 1904; Dutton et al., 1905; Dworkin et al., 1998, 2008). In more severe cases, infection with RF spirochetes can be associated with other manifestations including jaundice, meningitis, acute respiratory distress syndrome, and perinatal mortality (Jongen et al., 1997; Dworkin et al., 1998; Centers for Disease and Prevention, 2007). Tickborne RF (TBRF) is globally distributed with high prevalence in several endemic areas (Cutler, 2010). Accordingly, TBRF is the most common bacterial infection in Senegal and the most prevalent cause of fever in rural Zaire (Dupont et al., 1997; Vial et al., 2006). Moreover, the actual incidence of TBRF may be even higher than reported in many studies, as TBRF cases often go unreported or are misdiagnosed as another disease, such as malaria, in endemic regions of Africa (Dworkin et al., 1998; Nordstrand et al., 2007; Cutler, 2010; Schwan et al., 2012; Talagrand-Reboul et al., 2018). Despite the public health concern that TBRF represents, and the fact that the etiologic agent of TBRF was first described over 100 years ago (Ross and Milne, 1904; Dutton et al., 1905), our knowledge regarding virulence determinants utilized by the causative *Borrelia* spirochetes is limited.

RF and Lyme disease (LD) *Borrelia* are evolutionarily related spirochetes with chromosomes that are largely colinear (Hyde and Johnson, 1986; Fraser et al., 1997; Guyard et al., 2006; Lescot et al., 2008; Miller et al., 2013). In fact, comparison of the chromosomes of these two groups revealed only 17 genes unique to RF spirochetes and 13 genes unique to LD spirochetes (Lescot et al., 2008). Despite this similarity, TBRF and LD *Borrelia* have very disparate enzootic cycles and cause unique diseases. With respect to the enzootic cycle, most TBRF spirochetes are transmitted by *Ornithodoros* soft ticks, whereas LD spirochetes are spread by *Ixodes* hard ticks (Davis, 1936, 1941; Burgdorfer et al., 1982; Barbour, 2018; Barbour and Schwan, 2019). TBRF spirochetes colonize both the midgut and salivary glands of the *Ornithodoros* tick and are transmitted to the mammalian host within seconds of tick attachment (Schwan and Piesman, 2002; Boyle et al., 2014). Alternatively, LD spirochetes primarily colonize the tick midgut and migrate to the salivary glands only after the tick begins feeding (Ribeiro et al., 1987). Therefore, colonized *Ixodes* ticks must feed more than 24 h to transmit spirochetes (Piesman et al., 1987). During mammalian infection with TBRF spirochetes, characteristic recurring bacteremic episodes occur with spirochetes reaching numbers as high as 10^8^ bacteria/mL in the blood (Stoenner et al., 1982; Cadavid et al., 1994; Pennington et al., 1997; Dworkin et al., 1998, 2008; Cutler, 2010). Conversely, during mammalian infection by LD spirochetes, bacteremia primarily occurs early during infection and at relatively lower levels (10^3^-10^4^ bacteria/mL of blood), during which bacteria are disseminating to distal tissues (Wang et al., 2002). Therefore, whereas symptoms of TBRF are predominantly due to high amounts of bacteria present in the bloodstream, LD symptoms are often the result of specific tissue colonization. These differences suggest that TBRF and LD spirochetes have evolved to encode unique tick colonization factors and virulence factors essential for their distinct enzootic cycles and associated disease courses.

While the chromosomes of TBRF and LD *Borrelia* species are mostly colinear, their chromosomes also contain a subset of conserved genes that are only found in either TBRF or LD spirochetes (Hyde and Johnson, 1986; Fraser et al., 1997; Guyard et al., 2006; Lescot et al., 2008; Miller et al., 2013). It has been hypothesized that the chromosomal genes unique to TBRF or LD *Borrelia* may encode important bacterial factors that contribute to the distinct aspects of the enzootic cycles and disease courses of the two groups of *Borrelia* (Guyard et al., 2006). *bt0790A*, designated *btpA* in *Borrelia turicatae* by Guyard et al., encodes an HtrA (high temperature requirement) family serine protease found in the chromosomes of TBRF spirochetes, but not LD spirochetes (Guyard et al., 2006). The HtrA family of serine proteases are important for the pathogenesis of several species of bacteria, including *Salmonella typhimurium, Yersinia enterocolitica*, and *Listeria monocytogenes* (Johnson et al., 1991; Li et al., 1996; Pallen and Wren, 1997; Schafer et al., 1999; Clausen et al., 2002; Raivio, 2005; Wilson et al., 2006; Ingmer and Brondsted, 2009). Periplasmic HtrA proteases serve as stress-response chaperones that assist in polypeptide folding or as proteases involved in turnover of improperly folded proteins (Strauch and Beckwith, 1988; Spiess et al., 1999; Rizzitello et al., 2001). In light of this key physiological role, it is not surprising that *htrA* mutants of many bacteria are more sensitive to environmental stresses encountered during human infection that result in misfolded proteins, including increased temperature and oxidative stress (Lipinska et al., 1989; Johnson et al., 1991; Elzer et al., 1994; Li et al., 1996; Cortes et al., 2002; Brondsted et al., 2005; Wilson et al., 2006). Guyard et al. demonstrated that BhpA, the *Borrelia hermsii* ortholog of BtpA that shares 89% identity, has caseinlolytic activity, and is located intracellularly, presumably in the periplasm (Guyard et al., 2006). Guyard et al. also showed elevated transcription of *bhpA* when bacteria were cultured at mammalian body temperature relative to a temperature representative of an unfed tick, suggesting a possible role during mammalian infection. Finally, they demonstrated that heterologous expression of *bhpA* in *Borrelia burgdorferi*, a LD spirochete, rendered the bacteria more resistant to oxidative stress, and neutrophil-mediated killing *in vitro* (Guyard et al., 2006). It was therefore hypothesized that these unique proteases in TBRF spirochetes play a role in turnover of proteins damaged by the immune response [e.g., reactive oxygen species (ROS) and reactive nitrogen species (RNS)] and facilitate the capacity for these bacteria to achieve high-level bacteremia relative to LD spirochetes (Guyard et al., 2006).

Herein, we aimed to further the work of Guyard et al. (2006). Proteomic analyses revealed that, in *B. turicatae*, BtpA and proteins encoded by adjacent chromosomal genes are produced in response to growth at mammalian body temperature. Subsequent transcriptional analyses demonstrated that *btpA* is expressed in an operon with immediately upstream and downstream genes, which encode a hypothetical protein and thymidine kinase, respectively. Further, the analogous region of the *B. burgdorferi* chromosome was also co-transcribed, suggesting that the operonic nature of this region of the chromosome is conserved among diverse *Borrelia* species. Importantly, in the previous study, attempts to delete *bhpA* in *B. hermsii* were unsuccessful, leading to the suggestion that this protease may be required for bacterial viability (Guyard et al., 2006). However, we were able to generate mutants lacking *btpA* in the TBRF spirochete, *B. turicatae*, and directly assess the importance of this protease *in vitro* under environmental stresses, and *in vivo* during mammalian infection. *btpA* mutants did not exhibit increased susceptibility to high culture temperature like *htrA* mutants of several other bacteria. Furthermore, *btpA* mutants were equally resistant to oxidative stress produced by hydrogen peroxide (H_2_O_2_) and nitrosative stress produced by the nitric oxide donor diethylamine NONOate (DEA/NO) relative to wild-type *B. turicatae*, however, exhibited slightly increased susceptibility to *t*-butyl peroxide. Lastly, *in vivo* murine infection experiments demonstrated that mutants are able to establish initial bloodstream infection and cause subsequent bacteremic relapses. These data imply, contradictory to previous work, that BtpA is not required by TBRF spirochetes to facilitate mammalian infection.

## Materials and Methods

### Bacterial Strains and Culture Conditions

Bacterial strains and plasmids used in this study are detailed in Table 1. *Escherichia coli* strain TOP10F' (Life Technologies, Carlsbad, CA) was utilized for cloning. *E. coli* cultures were grown at 37°C in Luria-Bertani (LB) medium supplemented with 100 μg/mL ampicillin or 5 μg/mL gentamicin when necessary. Low passage strains of *B. turicatae* [strain 91E135 (Oz1)] and *B. burgdorferi* (strain 297) were used for this study (Taylor et al., 1991; Hughes et al., 1992; Schwan et al., 2005). Specifically, wild-type *B. turicatae* and *B. burgdorferi* were passaged no more than twice from the original frozen stock, and *B. turicatae btpA* mutants were passaged no more than twice after clonal isolation (see below). *B. turicatae* was cultured at 35°C with 3% CO_2_ in modified Barbour-Stoenner-Kelly (mBSK) medium with 12% rabbit serum at a pH of 7.6 (Barbour, 1984; Battisti et al., 2008), except during oxidative and nitrosative stress susceptibility assays (below). For *B. turicatae* growth temperature experiments, all culture conditions were identical to those above with the exception of temperature, which was increased to 37, 39, or 41°C. For the temperature shift experiment, culture conditions were identical to the parameters above, except cultures were incubated at 23°C for seven days, followed by a shift to 37°C. Selection of *B. turicatae* transformants was achieved by supplementing mBSK medium with 40 μg/mL gentamicin. *B. burgdorferi* was cultured at 35°C in 3% CO_2_ in BSK-II medium with 6% rabbit serum at a pH of 7.6 (Barbour, 1984; Pollack et al., 1993).

**Table 1:** Plasmids and strains used in this study.

**Plasmid or strain**	**Description^a^^,^^b^**	**Source**
**PLASMID**		
pGEM-T Easy	TA cloning vector; Amp^r^	Promega
pUAMS4	pGEM-T Easy::P*flgB*-*aacC1* (*Asc*I-flanked); Gent^r^, Amp^r^	This study
pUAMS169	pGEM-T Easy::*aacC1* (Flanked with 5′*NdeI* and 3′*AscI*); Gent^r^, Amp^r^	This study
pUAMS177A	*btpA*::P*flgB*-*aacC1* mutagenesis construct; Gent^r^, Amp^r^	This study
pUAMS238	*btpA*::*aacC1* mutagenesis construct; Gent^r^, Amp^r^	This study
**STRAIN**		
*B. turicatae* 91E135 (Oz1)	*B. turicatae* tick isolate	Taylor et al., 1991; Schwan et al., 2005
*B. burgdorferi* 297	*B. burgdorferi* human isolate	Hughes et al., 1992
*E. coli* TOP10F'	F′ [*lacI*^q^Tn*10*(Tet^r^)] *mcrA* Δ(*mrr-hsdRMS-mcrBC*) φ80*lacZ*ΔM15 *nupG* Δ*lacX74 recA1 ara*Δ*139* Δ(*ara-leu*)*7697 galU galK rpsL* (Str^r^) *endA1*	Life Technologies
*B. turicatae btpA*::P*flgB*-*aacC1*	*btpA* mutant with gentamicin resistance marker under transcriptional control of the *flgB* promoter	This study
*B. turicatae btpA*::*aacC1*	*btpA* mutant with promoterless gentamicin resistance marker	This study

a *Amp, ampicillin*.

b *Gent, gentamicin*.

### Reverse Transcription-PCR (RT-PCR) Analyses

RT-PCR was performed to evaluate possible transcriptional linkage of chromosomal segments spanning *bt0790*-*bt0791* in *B. turicatae*, and *bb0790*-*bb0791* in *B. burgdorferi*, as well as to assess transcription of *btpA* and adjacent genes. RNA was extracted as previously described (Blevins et al., 2007; Groshong et al., 2014). Briefly, cultures of *B. turicatae* or *B. burgdorferi* were grown to late exponential phase followed by treatment with 10% (vol/vol) RNA stop solution (Bernstein et al., 2002; Blevins et al., 2007). Cells were collected by centrifugation and stored at −80°C until RNA extraction was performed. Total RNA was isolated with TRIzol reagent (ThermoFisher Scientific, Waltham, MA), and purified with the RNeasy Mini Kit (Qiagen, Valencia, CA) per the manufacturer's instructions. RNA was then treated with RNase-free DNase I (Qiagen) to eliminate possible DNA contamination. Absence of contaminating genomic DNA (gDNA) was confirmed by PCR utilizing primers specific for an internal region of the *flaB* gene (*B. turicatae* primers: 5′ BtFlaB and 3′ BtFlaB; *B. burgdorferi* primers: 5′ Chrom and 3′ Chrom), and EmeraldAmp GT PCR Master Mix (TaKaRa Bio, Mountain View, CA). Primers used in this study are described in Table 2.

**Table 2:** Primers and probe used in this study.

**Primer designation**	**Sequence^a^^,^^b^**	**Purpose**
5′ BtFlaB	CTGGAATGGGTGTTGCAGGA	RT-PCR; PCR Screening
3′ BtFlaB	CTCCCTCTTGTTGTGCACCT	RT-PCR; PCR Screening
5′ Chrom	GATTATCAATCATAATACATCAGC	RT-PCR
3′ Chrom	TCTAAGCAATGACAAACATATTGG	RT-PCR
5′*bt0790*-*790A* link RT-PCR (P1—Figure 1)	CCCTGTTCGTTATGAAAATGCTTTGCTTGG	RT-PCR
3′*bt0790*-*790A* link RT-PCR (P2—Figure 1)	GCAAAGAAACTCGCAAGCATTGGGTCC	RT-PCR
5′*bt0790A*-*791* link RT-PCR (P3—Figure 1)	GCTCCTAATTCTCCTGCAGATATTGGG	RT-PCR
3′*bt0790A*-*791* link RT-PCR (P4—Figure 1)	GGCCTACATCAAAAGAATCACCTGC	RT-PCR
5′*bt0790A* ORF RT-PCR (P3—Figure 2)	TGTAAGACTTCCAAGAGGCAAGGG	RT-PCR; PCR Screening
3′*bt0790A* ORF RT-PCR (P4—Figure 2)	TTTCATTTACCACAGGTCCACCGG	RT-PCR; PCR Screening
5′*bt0790* ORF RT-PCR	GGGAAGTTTTAGTAAAGTGTTAGCCG	RT-PCR
3′*bt0790* ORF RT-PCR	ACTGCGGCATTATTTTTGTCATCTACA	RT-PCR
5′*bt0791* ORF RT-PCR_v2	CCATTACCAAGGGACGTAGAAA	RT-PCR
3′*bt0791* ORF RT-PCR_v2	ACAATCTCATCACCACCAACA	RT-PCR
5′*bb0790*-*bb0791* link (P5—Figure 1)	GGCTGCAATTTTAATAGTTTTGGGATC	RT-PCR
3′*bb0790*-*bb0791* link (P6—Figure 1)	CGTTCATCATAAAAACATGCCTCATC	RT-PCR
*bt0790* IDT SYBR FWD	ACTTGATTTACATGAGACTTGAAGC	qRT-PCR
*bt0790* IDT SYBR REV	AAAGAGTCGGCTAACACTTTACT	qRT-PCR
*bt0790A* IDT SYBR FWD	CCAATGCTTGCGAGTTTCTTT	qRT-PCR
*bt0790A* IDT SYBR REV	CCCATATCCACCCAAACTGTTA	qRT-PCR
*bt0791* IDT SYBR FWD	CCATTACCAAGGGACGTAGAAAC	qRT-PCR
*bt0791* IDT SYBR REV	TCACTTCCACCGCCTCTATAA	qRT-PCR
5′ Bt*flaB* SYBR/ABI	AAAAACAGCTGAAGAGCTTGGAAT	qRT-PCR
3′ Bt*flaB* SYBR/ABI	CACCCACATGTACTCTTAATGTCCAT	qRT-PCR
5′ F1 *bt0790A* KO	CACCTGATGAAGCTTATATGTTTTTTA	Mutagenesis Cloning
3′ F1 *bt0790A* KO_*Asc*I	**GGCGCGCC**CTCTACTCTCAGCAAGCATCATACC	Mutagenesis Cloning
5′ F2 *bt0790A* KO_*Asc*I	**GGCGCGCC**AATATTATTGAGAGTATTATAGAG	Mutagenesis Cloning
3′ F2 *bt0790A* KO_*BssH*II	**GCGCGC**TCCCTACCAACAAGATAATGATGCC	Mutagenesis Cloning
3′ F1 *bt0790A* KO_*Nde*I	**CATATG**CTCTCAAAAGCTAATTAATGTTATGATAG	Mutagenesis Cloning
5′ F2 *bt0790A* KO_*Asc*I_v2	**GGCGCGCC**GCTAAGGTCTTTGCAAATGGTCTTGGTG	Mutagenesis Cloning
5′ Bt*flgB*-*Asc*I	**GGCGCGCC**AGCACCCGGTAGCAAGTTAAAAAAATTTG	Mutagenesis Cloning
3′ Gent-*Asc*I	**GGCGCGCC**TTAGGTGGCGGTACTTGGGTCG	Mutagenesis Cloning
5′ PromLess Bt Gent	GGCGCGCCATAGAGGGT**CATATG**TTACGCAGCAGC	Mutagenesis Cloning
5′*aacC1* diag	GCAACGATGTTACGCAGCAG	PCR Screening
3′*aacC1* diag	GCATCACTTCTTCCCGTATGC	PCR Screening
5′ Bt0790A ext diag_V2 (P1—Figure 2)	GATAAAGGAGTTTTGAAAGTTAAGAAAG	PCR Screening
3′ Bt0790A ext diag_V2 (P2—Figure 2)	CATAAAGCAATAAGACAAACACTCTCT	PCR Screening
Bt*flaB* F	CCAGCATCATTAGCTGGATCAC	qPCR
Bt*flaB* R	GTTGTGCACCTTCCTGAGC	qPCR
Bt*flaB*-Probe	/5YakYel/TGCAGGTGA/ZEN/AGGTGCGCAGGTT/3IABkFQ/	qPCR

a *Relevant restriction sites are indicated by bold lettering*.

b *YakYel, 5′ Yakima Yellow dye; ZEN, ZEN internal quencher; IABkFQ, Iowa Black FQ 3′ quencher*.

Purified RNA was converted to cDNA using the iScript cDNA Synthesis Kit (Bio-Rad Laboratories, Hercules, CA) or SuperScript IV VILO Master Mix (ThermoFisher Scientific) according to the manufacturers' protocols. As a negative control, a mock reaction was conducted in the absence of reverse transcriptase. cDNA was then used as template for PCRs with EmeraldAmp GT PCR Master Mix and primers either annealing to adjacent genes to assess transcriptional linkage or primers annealing to internal regions of genes to evaluate transcription (Table 2). For the *bt0790*-*bt0791* RT-PCR linkage reaction however, PrimeSTAR Max DNA polymerase (TaKaRa Bio) was used in place of EmeraldAmp GT PCR Master Mix due to the need to generate a larger PCR product. Wild-type *B. turicatae* or *B. burgdorferi* gDNA was used as an amplification control where appropriate. PCR products were separated by gel electrophoresis in 0.8% agarose and stained with ethidium bromide for DNA visualization.

### Quantitative Real-Time PCR (qRT-PCR) Analyses

qRT-PCR was used to measure transcription of genes in the *btpA*-containing operon in *btpA* mutant spirochetes relative to wild-type *B. turicatae*. RNA was isolated and converted to cDNA as described above. Primers were designed to detect transcription of *bt0790* (primers: *bt0790* IDT SYBR FWD and *bt0790* IDT SYBR REV), *btpA* (primers: *bt0790A* IDT SYBR FWD and *bt0790A* IDT SYBR REV), and *bt0791* (primers: *bt0791* IDT SYBR FWD and *bt0791* IDT SYBR REV), as well as *flaB* (primers: 5′ Bt*flaB* SYBR/ABI and 3′ Bt*flaB* SYBR/ABI) as a control gene. SsoAdvanced Universal SYBR Green Supermix (Bio-Rad Laboratories) was used per the manufacturer's instructions. Briefly, a master mix was made so that 19 μL contained 10 μL of the above 2X supermix, 1 μL of each primer at a concentration of 10 μM, and 7 μL of nuclease free water. 19 μL aliquots of the master mix were then distributed into wells in a 96-well real-time PCR reaction plate. 1 μL of cDNA template at a concentration of 100 ng/μL was then added to the 19 μL of master mix in each well, resulting in a final primer concentration of 500 nM. To check for DNA contamination, no template control (NTC) reactions were conducted in which 1 μL of nuclease free water was added to the 19 μL of master mix instead of cDNA template. Reactions were performed using the QuantStudio 6 Flex Real-Time PCR System (ThermoFisher Scientific), and reaction conditions included an initial polymerase activation step at 95°C for 30 s, followed by 40 cycles of DNA denaturation at 95°C for 10 s and primer annealing/DNA extension at 60°C for 30 s. Three technical replicates were conducted for each experiment, and two biological replicates were performed. Data was analyzed using the ΔΔC_t_ method as previously described (Livak and Schmittgen, 2001).

### Proteomic Analysis

To evaluate temperature-dependent differences in *B. turicatae* protein production, wild-type *B. turicatae* cultures were inoculated at an initial density of 10^4^ bacteria/mL and grown at either 37°C or 23°C to the late exponential growth phase. Spirochetes were collected by centrifugation, washed two times in cold saline, and then prepared for SDS-PAGE. For each sample, a volume of whole cell lysates equivalent to approximately 3 × 10^7^ spirochetes was loaded in a 4–20% Mini-PROTEAN TGX gel (Bio-Rad Laboratories), and proteins were separated by electrophoresis. After SDS-PAGE, whole lanes were excised in slices, and subjected to in-gel trypsin digestion of proteins. Proteins were identified and quantified by LC-MS/MS based on spectral counts of tryptic peptides for the proteins of interest. Sample preparation, protein identification, and analysis was carried out by the UAMS Proteomics Core as previously described (Zielinska et al., 2012; Byrum et al., 2018). Proteins were then identified using Mascot version 2.5.1 (Matrix Science, Boston, MA) and the *B. turicatae* strain 91E315 database (GenBank assembly accession: GCA_000012085.2). Comparisons were performed on two biological replicates for 37°C cultures and three biological replicates for 23°C cultures.

### Generation of *btpA* Mutants

Allelic exchange mutagenesis was used to inactivate *btpA* in *B. turicatae* (Lopez et al., 2013). Primers used are described in Table 2. All PCRs for cloning were performed with high-fidelity PrimeSTAR Max DNA polymerase. The gentamicin resistance cassette, P*flgB-aacC1*, was generated by fusing the promoter for the *B. turicatae* flagellar basal body rod protein (*flgB*; *bt0294*) to the *aacC1* resistance marker and introducing AscI sites flanking the cassette (primers: 5′ Bt*flgB*-*Asc*I and 3′ Gent-*Asc*I) (Battisti et al., 2008). The resistance cassette was then TA-cloned into pGEM-T Easy (Promega Corp., Fitchburg, WI). The *btpA*::P*flgB*-*aacC1* mutational construct was generated by amplifying a 5′ flanking region (primers: 5′ F1 *bt0790A* KO and 3′ F1 *bt0790A* KO_*Asc*I), and a 3′ flanking region (primers: 5′ F2 *bt0790A* KO_*Asc*I and 3′ F2 *bt0790A* KO_*BssH*II). The respective amplicons were subsequently TA-cloned into pGEM-T Easy and sequence confirmed. The 5′- and 3′-flanking region fragments were ligated together with the P*flgB-aacC1* cassette between them to yield the final *btpA*::P*flgB*-*aacC1* mutational construct, pUAMS177A.

Derivation of the construct to inactivate *btpA* with a promoterless *aacC1* marker was similar to that described above. A 5′ flanking region (primers: 5′ F1 *bt0790A* KO and 3′ F1 *bt0790A* KO_*Nde*I) and a 3′ flanking region (primers: 5′ F2 *bt0790A* KO_*Asc*I_v2 and 3′ F2 *bt0790A* KO_*BssH*II) were PCR amplified (Table 2). The promoterless marker was generated by amplifying the *aacC1* open reading frame (ORF) from P*flgB-aacC1* with primers that introduced 5′ NdeI and 3′ AscI restriction sites (primers: 5′ PromLess Bt Gent and 3′ Gent-*Asc*I). The respective amplicons were subsequently TA-cloned into pGEM-T Easy and sequence confirmed. The 5′- and 3′-flanking region fragments were ligated together with the *aacC1* ORF between them to generate the final *btpA*::*aacC1* allelic exchange construct, pUAMS238. The *aacC1* ORF was introduced at the start codon of *bt0790A*.

Constructs were electroporated into wild-type *B. turicatae*, transformants were selected for with gentamicin, and clones were obtained via serial dilution plating as previously described (Lopez et al., 2013). Disruption of the gene in both mutants was genotypically confirmed using PCR with primers (Table 2) that amplify an internal segment of *btpA* (primers: 5′ Bt0790A ORF RT-PCR and 3′ Bt0790A ORF RT-PCR), a segment flanking the deleted region (primers: 5′ Bt0790A ext diag_V2 and 3′ Bt0790A ext diag_V2), an internal segment of the *aacC1* ORF (primers: 5′ *aacC1* diag and 3′ *aacC1* diag), and an internal segment of the *flaB* gene (primers: 5′ BtFlaB and 3′ BtFlaB) as a positive amplification control.

### Oxidative and Nitrosative Stress Susceptibility Assays

Low passage *B. turicatae* strains were grown to stationary phase (~2 × 10^8^ cells/mL) under aerobic conditions (5% CO_2_, 18% O_2_) at 34°C in modified pyruvate-free mBSK media (Troxell et al., 2014). Strains were pelleted by centrifugation and resuspended to a density of 1–2 × 10^7^ cells/mL in modified pyruvate-free mBSK media. One mL aliquots were transferred to 5-mL polypropylene culture tubes (Midwest Scientific, Valley Park, MO) and cultured in the presence or absence of H_2_O_2_, *t*-butyl peroxide, or diethylamine NONOate (Cayman Chemical, Ann Arbor, MI) for 2 h under aerobic conditions at 34°C. Following incubation, serial dilutions of the cultures were prepared in mBSK and were plated on semi-solid mBSK media with 12% rabbit serum in 6-well culture plates as described previously (Raffel et al., 2018). Colony forming units (CFUs) were enumerated after 8–10 days of incubation. Percent survival was determined by dividing CFUs from the 2 h timepoint samples by the CFUs from the 0 h timepoint. Data are presented as mean ± standard deviation (SD).

### Murine Infections

Murine infections were carried out in accordance with the recommendations of the Guide for the Care and Use of Laboratory Animals, the Public Health Science Policy on Humane Care and Use of Laboratory Animals, and the Animal Welfare Act. The protocol was approved by the University of Arkansas for Medical Sciences Institutional Animal Care and Use Committee (IACUC). Wild-type *B. turicatae* and *B. turicatae btpA* mutants were passaged no more than two times from the original frozen stocks. Groups of 4- to 6-week-old, female Swiss Webster mice (Charles River Laboratories, Wilmington, MA) were used in this study. Wild-type *B. turicatae, btpA*::P*flgB*-*aacC1*, and *btpA*::*aacC1* strains were grown to mid- to late-log phase, enumerated by dark-field microscopy, and diluted in fresh mBSK media to a concentration of 10^3^ spirochetes/mL. 100 μL of this dilution (containing 10^2^ total spirochetes) was then intradermally/subcutaneously injected into mice in the thoracic region. Daily blood samples, taken via tail venipuncture, were collected on days 3 to 14 post-infection for bacterial burden quantitative PCR (qPCR) assays as previously described (Mccoy et al., 2010; Boyle et al., 2014). 2.5 μL of blood was immediately combined with 47.5 μL of SideStep Lysis & Stabilization Buffer (Agilent Technologies, Santa Clara, CA) and stored at −80°C (Mccoy et al., 2010; Boyle et al., 2014). For preparation of qPCR standard curve samples, described below, blood was collected from uninfected female Swiss Webster mice by brachial artery bleed and combined with SideStep Lysis & Stabilization Buffer at a blood-to-buffer ratio of 1:18.

### qPCR for Bacterial Burdens

TaqMan-based qPCR analysis was conducted as initially described by McCoy et al. for *B. hermsii* (Mccoy et al., 2010) and Boyle et al. for *B. turicatae* (Boyle et al., 2014), but with some modifications. 17 μL of master mix containing the following components was added to a 96-well real time PCR plate; 10 μL 2x SsoAdvanced Universal Probes Supermix (Bio-Rad Laboratories), 0.8 μL of each forward and reverse primer at a concentration of 10 μM (primers: Bt*flaB* F and Bt*flaB* R), 1.2 μL of probe at a concentration of 5 μM [probe: Bt*flaB* probe labeled with 5′ Yamika Yellow and double-quenched with an internal ZEN quencher and 3′ Iowa Black FQ quencher (Integrated DNA Technologies, Coralville, IA)], and 4.2 μL of nuclease free water. 3 μL of the blood sample in lysis/stabilization buffer (see above) was then added to each well; resulting in a final concentration of 400 nM for each primer and 300 nM for the probe. Samples for standard curves were generated by centrifuging 1 mL of late-exponential phase *B. turicatae* culture at 6,000 × g for 15 min at room temperature. Supernatant was discarded and cells were resuspended in 1 mL of phosphate-buffered saline with 5 mM MgCl_2_ (PBS-MgCl_2_). This wash step was then repeated two more times. Following the final centrifugation, spirochetes were resuspended in 500 μL PBS-MgCl_2_ and enumerated by dark-field microscopy. This suspension was then diluted with PBS-MgCl_2_ to a density of 10^8^ spirochetes/mL, and a range of 10-fold serial dilutions were prepared from 10^4^ to 10^8^ spirochetes/mL. For purposes of a NTC, nuclease free water was diluted 10-fold in PBS-MgCl_2_. Preparations from 10^4^ to 10^8^ spirochetes/mL and the NTC were spiked into naïve blood in lysis/stabilization buffer (see above) at a ratio of 1:19. 3 μL of this final preparation were added to 17 μL of the above master mix and used to generate a standard curve for qPCR. All sample and standard curve reactions were conducted in triplicate. Real-time qPCR was performed using the QuantStudio 6 Flex Real-Time PCR System (ThermoFisher Scientific). The run method consisted of an initial 50°C hold for 2 min followed by a polymerase activation step at 95°C for 10 min. DNA amplification was performed by running 40 cycles consisting of DNA denaturation at 95°C for 15 s and primer annealing/DNA extension at 60°C for 60 s. Data obtained was subsequently imported into Prism version 6 (GraphPad Software, San Diego, CA), graphed, and analyzed.

### Statistical Methods

To compare maximum bacterial burdens in the blood of infected mice (qPCR results) and survival following exposure to ROS/RNS, analysis of variance (ANOVA) models were used with Tukey's procedure for pairwise comparisons. *p* < 0.05 were considered statistically significant. For qRT-PCR analyses, mean and standard error of the mean (SEM) for fold-change was calculated based on the ΔΔC_t_ values before log-transformation (i.e., evaluating the 2^−ΔΔCt^ term) as previously described (Livak and Schmittgen, 2001). Statistical tests were performed using Prism version 6 (GraphPad Software).

## Results

### BtpA Is Produced in Response to Mammalian Body Temperature

In the LD spirochete, *B. burgdorferi*, numerous proteins important for mammalian infection are produced when the bacteria are cultured at mammalian body temperature (37°C), whereas, proteins involved in tick colonization are produced in response to culture at a temperature representative of an unfed tick (23°C) (Schwan et al., 1995; Stevenson et al., 1995; Schwan and Piesman, 2000; Yang et al., 2000; Ojaimi et al., 2003). Guyard et al. demonstrated that, in *B. hermsii, bhpA* transcription is significantly higher at 37°C relative to 23°C, suggesting a potential role for BhpA during mammalian infection. Additionally, Guyard et al. showed production of BhpA at 23, 34, and 37°C by immunoblot, but did not quantify possible temperature-dependent differences (Guyard et al., 2006). We therefore sought to assess whether production of BtpA in *B. turicatae* was elevated at 37°C relative to 23°C. To this end, wild-type *B. turicatae* was cultured at these two temperatures and bacterial proteins were separated by SDS-PAGE. Gel slices were prepared from the lanes and proteins in each slice were identified and quantified via LC-MS/MS. Results for the proteins encoded by *btpA* and adjacent genes are presented in Table 3. BtpA-derived peptides were only detected at 37°C and not 23°C, supporting the hypothesis that BtpA plays a potential role during mammalian infection. Similarly, BT0790 and BT0791, a hypothetical protein and thymidine kinase, respectively, were produced at ~3-fold more at mammalian body temperature relative to 23°C. In contrast, BT0789 and BT0792 were produced at similar levels between these two temperatures. These results agree with the transcriptional results of Guyard et al. (2006), and suggest that BtpA, as well as BT0790 and BT0791, may be important for mammalian infection.

**Table 3:** Impact of cultivation temperature on production of BtpA and proteins encoded by adjacent chromosomal genes in *B. turicatae*.

**Protein**	**23°C average spectral counts (± SEM)**	**37°C average spectral counts (± SEM)**	**Fold change in production (37°C/23°C spectral counts)**	**Identity**
BT0789	104.30 ± 4.92	83.80 ± 1.58	0.80	FtsH family protease
BT0790	2.16 ± 0.09	6.62 ± 0.13	3.06	Hypothetical protein
BT0790A (BtpA)	0.00 ± 0.00	3.85 ± 0.48	>3.85^*^	HtrA family serine protease
BT0791	0.67 ± 0.67	3.33 ± 1.17	4.97	Thymidine kinase
BT0792	1.49 ± 0.75	1.10 ± 0.02	0.74	Hypothetical protein

* *Only detected at 37°C*.

*SEM; Standard error of the mean*.

### *btpA* Is Transcribed in an Operon

**Figure 1:**
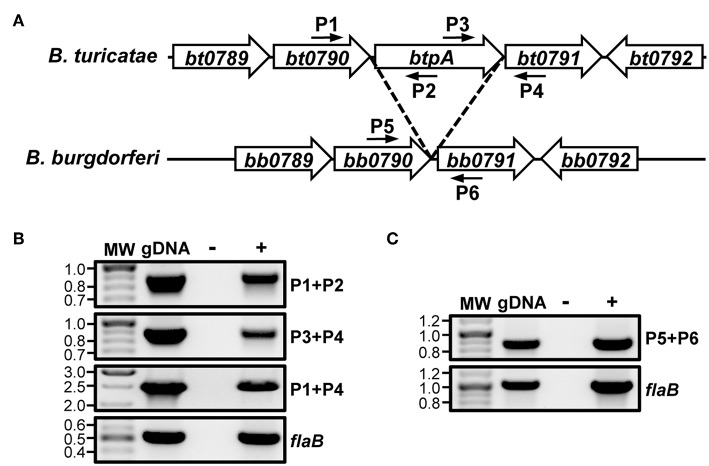
*btpA* is transcriptionally linked to *bt0790* and *bt0791*. **(A)** Selected regions of *B. turicatae* and *B. burgdorferi* chromosomes; adapted from Guyard et al. (2006). Numbered smaller arrows indicate primers used in **(B,C)**. **(B)** Transcriptional linkage of *btpA* to *bt0790* and *bt0791* in *B. turicatae*. RNA was isolated from wild-type *B. turicatae* culture and converted to cDNA (+). Mock reactions without reverse transcriptase were performed as a negative control (–). Wild-type *B. turicatae* genomic DNA (gDNA) was included as a positive control for each reaction. Approximate locations of primers in the genome are indicated by smaller arrows and numbers in **(A)**. PCRs were performed to amplify intergenic regions between genes within the suspected operon: *bt0790-btpA* (P1+P2, 886 bp)*, btpA-bt0791* (P3+P4, 887 bp), and *bt0790-bt0791* (P1+P4, 2,527 bp). Another PCR was performed to amplify an internal region of *flaB* (519 bp). MW denotes the DNA standard, and numbers to the left indicate molecular weights in kb. **(C)** Transcriptional linkage of *bb0790* and *bb0791* in *B. burgdorferi*. RNA was isolated from *B. burgdorferi* culture and converted to cDNA (+). Mock reactions without reverse transcriptase were performed as a negative control (–). *B. burgdorferi* genomic DNA (gDNA) was included as a positive control for each reaction. Approximate locations of primers in the genome are indicated by smaller arrows and numbers in **(A)**. A PCR was performed to amplify an intergenic region between *bb0790-bb0791* (P5+P6, 861 bp). Another PCR was performed to amplify an internal region of *flaB* (1,006 bp). MW denotes the DNA standard, and numbers to the left indicate molecular weights in kb.

Analysis of the *B. turicatae* genome annotation revealed that the *btpA* ORF is encoded on the same DNA strand as *bt0790* and *bt0791*. Additionally, *btpA* is located 14 bp downstream of *bt0790* and overlaps 34 bp with *bt0791* (Hyde and Johnson, 1986; Fraser et al., 1997; Penningon et al., 1999; Guyard et al., 2006; Miller et al., 2013). Due to the proximity of *btpA* to the immediately adjacent genes and similar protein production patterns of BT0790, BtpA, and BT0791 (higher production at 37°C relative to 23°C), it was hypothesized that *bt0790, btpA*, and *bt0791* may be in an operon. To test this, RT-PCR was performed with primers designed to amplify across intergenic regions of adjacent ORFs within the potential operon (Figure 1A), as well as to amplify an internal region of *flaB* as a positive RT control. RT-PCR for *flaB* generated an amplicon of appropriate size, confirming successful RNA isolation and cDNA synthesis from *in vitro-*grown *B. turicatae* (Figure 1B). Amplification products were also obtained from reactions to assess linkage of *bt0790* to *btpA* and *btpA* to *bt0791*. Furthermore, an amplification product was observed linking *bt0790* to *bt0791*, indicating that *btpA* is transcribed with the immediately upstream (*bt0790*) and downstream (*bt0791*) genes.

Because of the colinear nature of *Borrelia* chromosomes, we hypothesized that the analogous region of LD spirochete genomes would also be co-transcribed. To test this, RT-PCR analyses were performed using *B. burgdorferi*. Reactions were conducted to amplify across the intergenic region between *bb0790* and *bb0791*, as well as an internal region of *flaB* as a positive control (Figure 1C). RT-PCR results indicated transcriptional linkage of *bb0790* and *bb0791*, suggesting that the operonic nature of this region of the chromosome is conserved among diverse *Borrelia* species.

### Inactivation of *btpA* in *B. turicatae*

**Figure 2:**
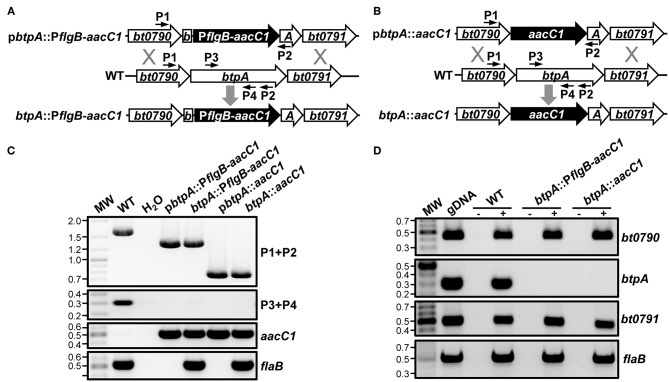
Generation of *B. turicatae btpA* mutants. **(A)** Organization of *btpA*::P*flgB*-*aacC1* mutant. A segment of *btpA* in wild-type *B. turicatae* (WT) was replaced with the *aacC1* gentamicin resistance marker expressed from the *B. turicatae flgB* promoter (*btpA*::P*flgB*-*aacC1*) via allelic exchange. **(B)** Organization of *btpA*::*aacC1* mutant. A segment of *btpA* in wild-type *B. turicatae* (WT) was replaced with a promoterless *aacC1* gentamicin resistance marker via allelic exchange. **(C)** Genotypic confirmation of *btpA* mutants. PCRs were conducted to amplify a region flanking the insertion site of the *aacC1* marker in *btpA* (P1+P2; WT, 1,649 bp; *btpA*::P*flgB*-*aacC1*, 1,319 bp; or *btpA*::*aacC1*, 745 bp) or internal segments of *btpA* (P3+P4; 305 bp), *aacC1* (689 bp), and *flaB* (519 bp). PCRs were conducted using genomic DNA (gDNA) from wild-type *B. turicatae* (WT), *btpA*::P*flgB*-*aacC1*, and *btpA*::*aacC1*, as well as the plasmids used to generate the mutants. PCRs were also conducted with no DNA as a purity control (H_2_O). MW denotes the DNA standard, and numbers to the left indicate molecular weights in kb. **(D)** Transcriptional confirmation of *btpA* mutants. RNA was isolated from wild-type *B. turicatae* (WT), *btpA*::P*flgB*-*aacC1*, and *btpA*::*aacC1* culture and converted to cDNA (+). Mock reactions without reverse transcriptase were performed as a control (–). Wild-type *B. turicatae* genomic DNA (gDNA) was included as a positive control for each reaction. PCRs were conducted with cDNA to amplify internal regions of *bt0790* (450 bp)*, btpA* (305 bp), *bt0791* (505 bp), and *flaB* (519 bp). MW denotes the DNA standard, and numbers to the left indicate molecular weights in kb.

Because Guyard et al. were unable to inactivate *bhpA* in *B. hermsii*, they could not directly test their hypothesis that BhpA is important for mammalian infection (Guyard et al., 2006). Their inability to mutate *bhpA* also led them to hypothesize that this protease might be required for bacterial viability. Contrary to the findings of Guyard et al., we were able to mutate *btpA* using two different allelic exchange-based mutational approaches (Lopez et al., 2013). The *btpA*::P*flgB*-*aacC1* mutant was made by replacing a 1012-bp internal region of the *btpA* ORF with an *aacC1* gentamicin resistance gene under transcriptional control of the promoter for the flagellar basal body rod protein (*flgB*) (Figure 2A). Considering that *btpA* is encoded in an operon, this mutational approach has the potential to be problematic as the *flgB* promoter could cause overexpression of the thymidine kinase gene, *bt0791*, that is downstream of *btpA*. To our knowledge, detrimental effects due to overexpression of a native thymidine kinase in prokaryotes has not been reported. However, to circumvent potential complications, an alternative mutational construct was also employed to generate a second independent *btpA* mutant. In this construct, designated *btpA*::*aacC1*, a promoterless *aacC1* marker was fused at the start codon of the *btpA* ORF to replace 1,446 bp of the 1,641-bp coding region (Figure 2B). In the *btpA*::*aacC1* mutant, the *aacC1* resistance gene is under transcriptional control of the native promoter of the *bt0790-bt0791* operon, thus reducing the possibility of detrimental polar mutation effects. Genotypic confirmation of the *btpA* mutants was performed by PCR amplifying a chromosomal region flanking the inserted antibiotic resistance marker, as well as internal regions of *btpA, aacC1*, and *flaB* (a positive control gene) (Figure 2C). In both *btpA* mutants, amplicons of the expected sizes were observed, and primers specific for an internal region of *btpA* only generated an amplicon with gDNA for wild-type *B. turicatae*. In addition, the *aacC1* marker was only detected in the *btpA* mutants. RT-PCR analyses were also conducted to confirm both the absence of *btpA* transcription in mutants and that the mutational strategies utilized did not significantly affect expression of other genes in the *bt0790-bt0791* operon (Figure 2D). In wild-type *B. turicatae, bt0790, btpA*, and *bt0791* transcription was readily detectable. As expected in both mutants however, *btpA* transcript was absent, whereas *bt0790* and *bt0791* were expressed. Successful generation of *btpA* mutants indicates that BtpA is not required for TBRF spirochete viability, as was previously hypothesized (Guyard et al., 2006), and allowed us to directly assess the importance of this protease in *B. turicatae* resistance to environmental stresses and during mammalian infection.

**Figure 3:**
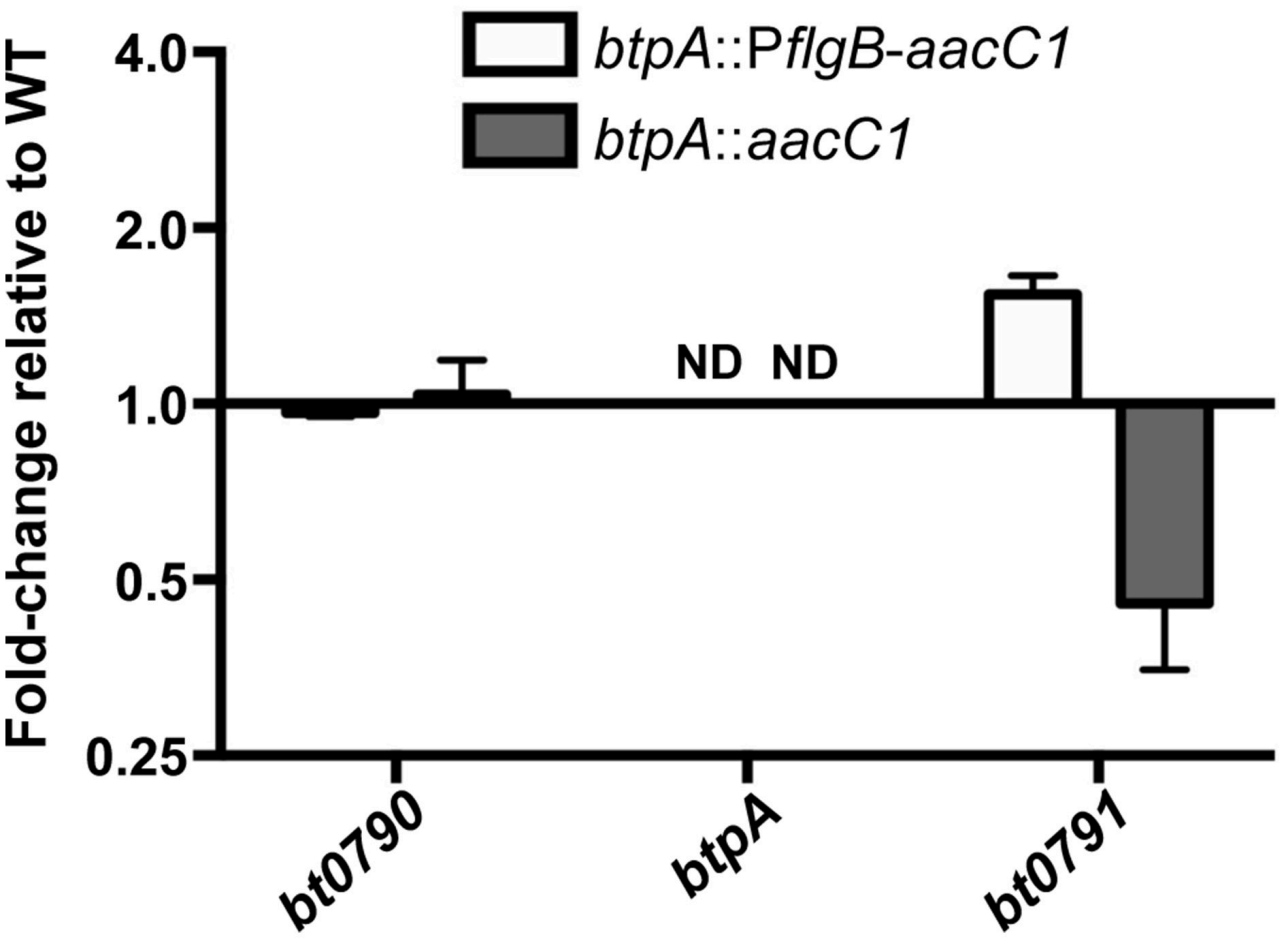
*btpA* mutants exhibit modest changes in *bt0791* expression. cDNA was generated from *in vitro* cultured wild-type, *btpA*::P*flgB*-*aacC1*, and *btpA*::*aacC1 B. turicatae* RNA and used for qRT-PCR analyses to determine possible expression changes in *bt0790, btpA*, and *bt0791*. Displayed are results from two independent biological replicates, and error bars represent standard error of the mean (SEM) with respect to fold-change values. Not detected, ND.

Because *btpA* is transcribed as part of an operon, and RT-PCR is only semi-quantitative, qRT-PCR analyses were conducted in order to detect possible polar effects associated with mutation of *btpA*. To this end, RNA was isolated from wild-type, *btpA*::P*flgB*-*aacC1*, and *btpA*::*aacC1* strains, converted to cDNA, and expression of genes in the *btpA-*containing operon (*bt0790, btpA*, and *bt0791*) was quantified (Figure 3). As expected, *btpA* expression was not detected in *btpA* mutants. In addition, expression of *bt0790* was similar between wild-type, *btpA*::P*flgB*-*aacC1* (mean = 0.96; SEM = 0.87–1.06), and *btpA*::*aacC1* (mean = 1.02; SEM = 0.79–1.32) strains. However, expression of *bt0791* was increased approximately 1.53-fold (SEM = 1.40–1.68) in the *btpA*::P*flgB*-*aacC1* mutant, consistent with our hypothesis that the P*flgB* promoter may drive overexpression of this downstream gene. In contrast, in the *btpA*::*aacC1* mutant, a 0.44-fold (SEM = 0.41–0.47) decrease in expression relative to wild-type *B turicatae* was observed. These observations suggest that subtle polar mutations were introduced with regard to *bt0791* expression via both mutational strategies. Therefore, it was important that the following experiments assessing *in vitro* and *in vivo* phenotypes associated with loss of *btpA* were conducted using both mutants in case slightly increased or decreased expression of *bt0791* in *btpA*::P*flgB*-*aacC1* and *btpA*::*aacC1* strains, respectively, could account for any phenotypes observed.

### Sensitivity to High Temperature Is Not Altered in *btpA* Mutants

**Figure 4:**
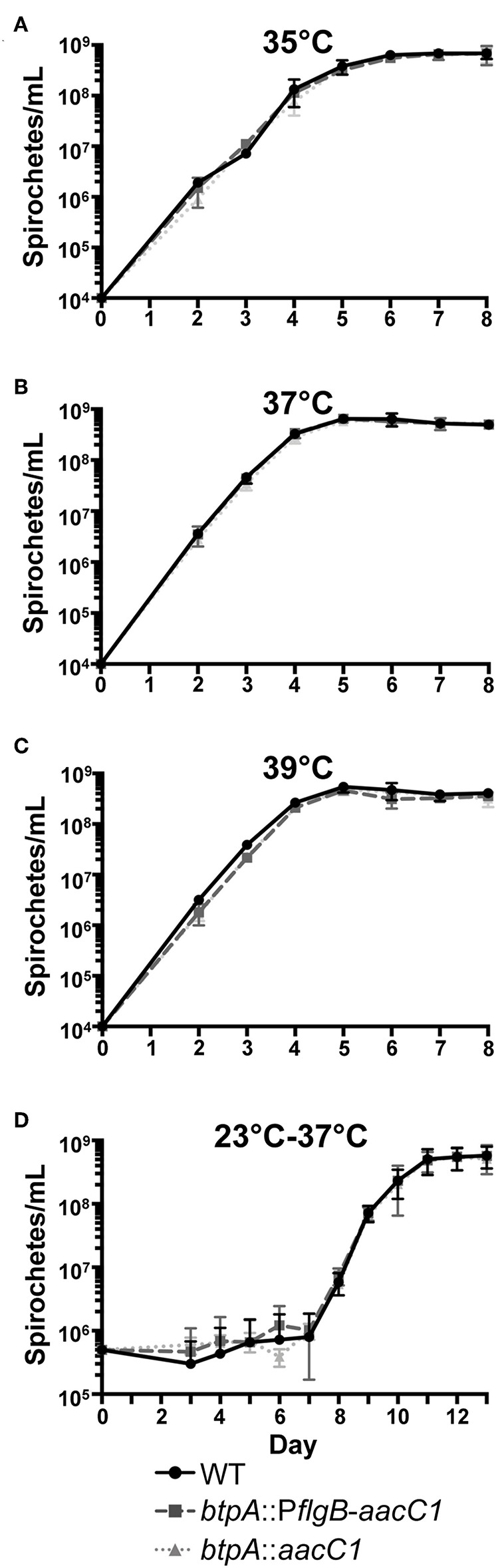
*btpA* mutants do not exhibit attenuated growth at elevated temperature. **(A–C)** *B. turicatae* wild-type (WT), *btpA*::P*flgB*-*aacC1*, and *btpA*::*aacC1* cultures were inoculated at an initial density of 10^4^ spirochetes/mL, grown at **(A)** 35°C, **(B)** 37°C, or **(C)** 39°C, and then counted daily by dark-field microscopy beginning on day 2 post-inoculation. **(D)** *B. turicatae* wild-type (WT), *btpA*::P*flgB*-*aacC1*, and *btpA*::*aacC1* cultures were inoculated at an initial density of 5 × 10^5^ spirochetes/mL at 23°C and then counted daily by dark-field microscopy beginning on day 3 post-inoculation. On day 7, cultures were shifted to a temperature of 37°C. Displayed are the results of two independent biological replicates, and error bars represent SEM.

High temperature requirement (HtrA) family proteases are designated as such because *htrA* mutants in *E. coli* exhibit a decreased growth rate when cultured at elevated temperatures (Lipinska et al., 1989). Subsequently, *htrA* mutants of several other bacteria, including *Y. enterocolitica, Campylobacter jejuni*, and *Brucella abortus*, were found to possess similar growth defects (Elzer et al., 1994; Li et al., 1996; Brondsted et al., 2005). To first evaluate whether *btpA* is required for adaptation of *B. turicatae* to elevated culture temperatures, wild-type, *btpA*::P*flgB*-*aacC1*, and *btpA*::*aacC1 B. turicatae* strains were grown at standard culture temperature (35°C), as well as two higher temperatures (Figures 4A–C), and counted daily. At the normal growth temperature, *btpA* mutants had similar growth to wild-type *B. turicatae*. Interestingly, *btpA* mutants also grew comparable to wild-type bacteria at 37 and 39°C. However, when we attempted to grow all strains at temperatures exceeding 39°C, no growth was observed (data not shown). To evaluate whether the *btpA* mutants exhibit a growth defect in response to the environmental temperature changes experienced by *B. turicatae* upon transition from tick to mammal, an experiment was conducted in which wild-type and *btpA* mutant strains were cultured at 23°C before being shifted to 37°C. Specifically, cultures were inoculated at an initial density of 5 × 10^5^ bacteria/mL, incubated at 23°C for seven days, and then shifted to a temperature of 37°C. *B. turicatae* cultured at 23°C do not demonstrate significant growth. Therefore, a higher inoculation density was required to monitor bacterial concentration and viability by dark-field microscopy at 23°C prior to culture at 37°C. In these growth comparisons, both wild-type *B. turicatae* and the *btpA* mutants demonstrated minimal growth at 23°C. Upon shifting the cultures to 37°C, the two *btpA* mutants and wild-type parent grew similarly (Figure 4D), indicating that *btpA* is not required by *B. turicatae* to survive environmental temperature shifts encountered during tick-to-mammal transmission. Collectively, these results suggest that, unlike HtrA family homologs in other bacterial species, BtpA is not required for resistance to heat shock *in vitro*.

### *btpA* Confers Resistance to Oxidative Stress Produced by *t*-butyl Peroxide

**Figure 5:**
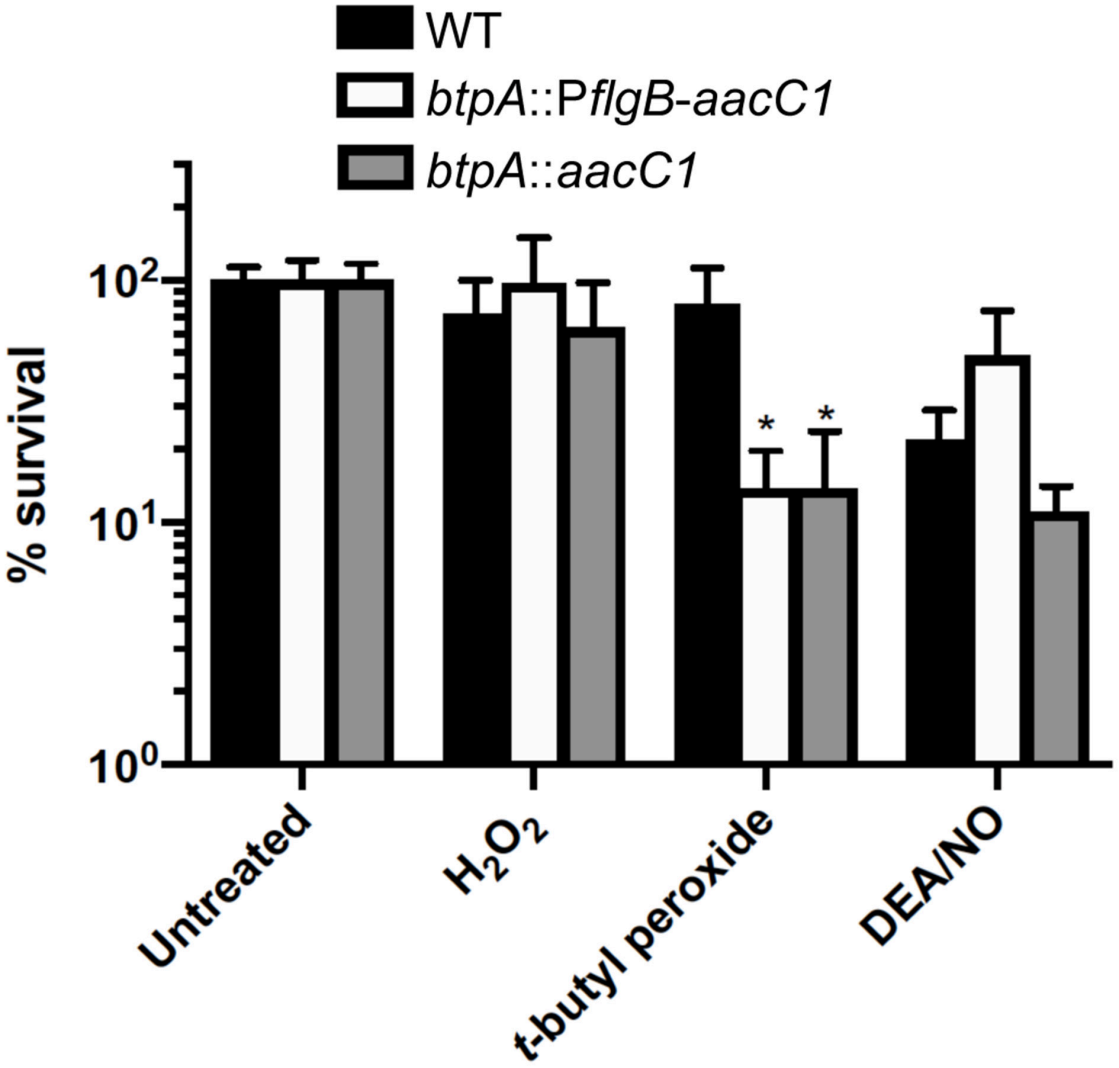
*btpA* contributes to resistance to *t*-butyl peroxide. *B. turicatae* strains were cultured in pyruvate-free mBSK in the presence or absence of 0.25 mM H_2_O_2_, 2.5 mM *t*-butyl peroxide, or 1.25 mM diethylamine NONOate (DEA/NO) for 2 h under aerobic conditions (5% CO_2_, 18% O_2_). Following treatment, serial dilutions were prepared in mBSK with 7 mM sodium pyruvate and plated on solid mBSK media. Data represent the mean percent survival ± standard deviation (SD) of 4 to 6 biological replicates. *p < 0.05 compared to wild-type *B. turicatae*.

Guyard et al. demonstrated that heterologously expressing *bhpA* in the LD spirochete, *B. burgdorferi*, increased its resistance to oxidative stresses in the form of *t*-butyl peroxide and diamide (Guyard et al., 2006). This finding led them to conclude that BhpA and orthologous proteins of TBRF spirochetes could play a role in resistance to oxidative stresses faced during bloodstream infection. Therefore, we hypothesized that *btpA* mutants would be more susceptible to ROS/RNS. To assess this hypothesis, the susceptibility of wild-type, *btpA*::P*flgB*-*aacC1*, and *btpA*::*aacC1 B. turicatae* strains to killing by H_2_O_2_, *t*-butyl peroxide, and the NO donor diethylamine NONOate (DEA/NO) were compared. All three strains exhibited similar levels of survival following challenge with 0.25 mM H_2_O_2_ and 1.25 mM DEA/NO (Figure 5). In contrast, exposure of cultures to 2.5 mM *t*-butyl peroxide resulted in an ~6-fold decrease in survival of the *btpA*-deficient *B. turicatae* strains (Figure 5). This latter finding is consistent with the previously described role of *bhpA* in defense against oxidative stress (Guyard et al., 2006).

### *btpA* Is Not Required for Mammalian Infection

**Figure 6:**
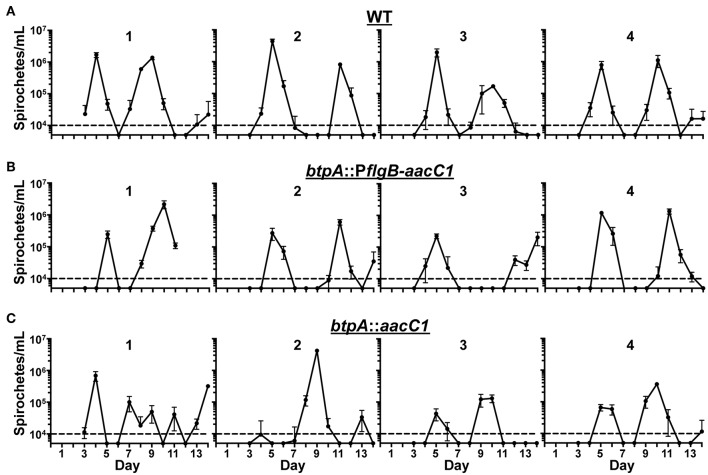
*btpA* is dispensable for mammalian infection. Groups of four mice were inoculated with a dose of 10^2^ spirochetes of the following strains; **(A)** wild-type (WT), **(B)** *btpA*::P*flgB-aacC1*, and **(C)** *btpA*::*aacC1*. Blood was collected daily between days 3 and 14, and spirochetemic burdens were then quantified by qPCR. Numbers above the graphs identify the mouse in each of the experimental groups. The dashed line represents the limit of detection of this assay (10^4^ spirochetes/mL), and error bars represent SEM.

We hypothesized that BtpA would be important for resistance to environmental stresses based on previous work (Guyard et al., 2006). However, we were unable to detect a growth defect in *btpA* mutants after incubation at increased culture temperature and only observed a slight increase in the sensitivity of the *btpA* mutants to oxidative stress produced by *t*-butyl peroxide. Although the *in vitro* defects observed in the *btpA* mutants were relatively modest, BtpA may still be important for survival of the multiple, simultaneous stresses the bacteria face during mammalian infection. To assess if BtpA is required for both initial bacteremia and subsequent relapses, a murine model of RF was utilized. Mice were intradermally inoculated with 100 spirochetes and infection was allowed to proceed until the mice were sacrificed at day 14. This dose was selected because 100 spirochetes is the lowest challenge dose of wild-type *B. turicatae* with which we are able to consistently establish infection in mice (data not shown). This timing allows for detection of the initial peak in spirochetemia (approximately days 4–6), as well as at least one subsequent relapse. Blood samples were collected daily, and bloodstream bacterial burden was quantified by qPCR (Figure 6). In both WT and *btpA* mutant strains, the maximum bacterial burden was 10^5^-10^7^ bacteria/mL. Additionally, relapse kinetics in WT and mutant strains were similar, with initial bacteremic peaks occurring between days 4–6 and first relapse occurring before day 12. Although the overall maximum bacterial burdens in the mice infected with the *btpA* mutants do appear to be slightly lower by comparison to the mice infected with WT parent, these differences were not statistically significant. Based on these infection results, *btpA* is not required for initial infection or subsequent bacterial relapse, suggesting that *btpA* is dispensable for the mammalian phase of the *B. turicatae* enzootic cycle.

## Discussion

Although RF represents a significant public health issue worldwide (Dupont et al., 1997; Vial et al., 2006; Talagrand-Reboul et al., 2018), very little information exists regarding specific virulence determinants that RF *Borrelia* spirochetes require to navigate their enzootic cycle. Guyard et al. proposed that an HtrA family protease (BtpA and orthologs) unique to TBRF spirochetes may serve an essential function during mammalian infection (Guyard et al., 2006). In support of this hypothesis, Guyard et al. demonstrated that *bhpA* was more highly expressed when *B. hermsii* was cultured at mammalian body temperature. To confirm these results in *B. turicatae*, we conducted a proteomic analysis with wild-type *B. turicatae* cultured at 37°C and 23°C to identify differences in protein production. BtpA was only detected at 37°C and not 23°C, consistent with the *bhpA* expression results. In addition, proteins encoded by genes immediately adjacent to *btpA* were also produced more than 3-fold more at mammalian body temperature relative to 23°C, leading us to hypothesize that *btpA* was co-transcribed with *bt0790* and *bt0791*. RT-PCR analyses confirmed this hypothesis. It should be noted that there are two caveats associated with this conclusion. First, transcriptional patterns do not always directly correlate with protein levels. Yet, the failure to detect BtpA at 23°C, while detecting BT0790 and BT0791, could suggest that these three genes don't comprise an operon. However, there were low average spectral counts obtained for BT0790, BtpA, and BT0791 at 23°C (2.16, 0.00, and 0.67, respectively). Thus, the absence of BtpA is likely due to protein levels at 23°C approaching the limit of detection for the analysis. Importantly though, BT0790, BtpA, and BT0791 were consistently detected at 37°C, which agreed with the prior transcriptional studies (Guyard et al., 2006). Second, replacing a portion of the *btpA* ORF with a promoterless antibiotic resistance marker appeared to decrease transcription of *bt0791*. This observation could indicate that an independent promoter, which is partly responsible for transcription of *bt0791*, exists within the region of the *btpA* ORF that was also disrupted in the *btpA*::*aacC1* mutant. Nonetheless, we still observed transcriptional linkage between *btpA* and *bt0791* in addition to the regulatory trends seen in temperature-dependent protein production; again, the latter agreed with the prior transcriptional studies (Guyard et al., 2006). Further studies are required to determine if a second alternative promoter also controls transcription of *bt0791*. Interestingly, we found that the operonic content of the region of the chromosome encoding *bt0790*-*bt0791* is conserved in the colinear *B. burgdorferi* chromosome. Though these are divergent *Borrelia* species, transcriptional similarities may imply conserved regulatory mechanisms. Accordingly, differential regulation in response to culture temperature has been noted in both LD and TBRF spirochetes (Schwan et al., 1995; Stevenson et al., 1995; Schwan and Hinnebusch, 1998; Schwan and Piesman, 2000; Yang et al., 2000; Revel et al., 2002; Ojaimi et al., 2003; Guyard et al., 2006; Marcsisin et al., 2012; Wilder et al., 2016; Neelakanta et al., 2017). However, the regulatory pathways mediating temperature-dependent changes in gene regulation in TBRF spirochetes remain unknown.

Guyard et al. was unable to directly test the hypothesis that the BtpA homolog of *B. hermsii*, BhpA, was important for resistance to environmental stresses *in vitro* or for mammalian infection due to an inability to generate a *bhpA* mutant (Guyard et al., 2006). In the LD spirochete, *B. burgdorferi*, molecular genetics are commonly used as a tool to assess the roles of proteins during the enzootic cycle (Rosa et al., 2005; Groshong and Blevins, 2014; Drecktrah and Samuels, 2018). However, as TBRF is relatively understudied, genetic manipulation of TBRF spirochetes is less frequently published. In fact, at the time of publication of the findings by Guyard et al., successful genetic manipulation of TBRF spirochetes had yet to be reported (Guyard et al., 2006; Battisti et al., 2008; Fine et al., 2011; Lopez et al., 2013). Moreover, genetic manipulation in TBRF spirochetes is still in its infancy, as the *btpA* mutants reported herein represent just the third publication of targeted mutagenesis in *B. turicatae* and the eighth publication utilizing targeted mutagenesis in all TBRF spirochetes (Battisti et al., 2008; Fine et al., 2011, 2014; Lopez et al., 2013; Raffel et al., 2014; James et al., 2016; Krishnavajhala et al., 2017). Additionally, the *btpA*::*aacC1* mutant is the first published use of a promoterless resistance marker for a mutagenesis approach in TBRF spirochetes. Using newly established means of genetically manipulating *B. turicatae*, we were able to extend the work of Guyard et al. and directly test whether BtpA is an important virulence factor in TBRF spirochetes mediating resistance to stresses faced during mammalian infection.

Based on phenotypes associated with *htrA* mutation in other bacteria and heterologous expression experiments performed by Guyard et al., we hypothesized that *btpA* mutants would exhibit increased sensitivity to heat shock and oxidative stress (Lipinska et al., 1989; Johnson et al., 1991; Elzer et al., 1994; Li et al., 1996; Cortes et al., 2002; Brondsted et al., 2005; Guyard et al., 2006; Wilson et al., 2006). However, no differences in growth were identified when the *btpA* mutants were cultured at higher temperatures. Interestingly though, we found that *B. turicatae* is unable to grow *in vitro* at temperatures exceeding 39°C. This differs from *B. burgdorferi sensu lato* strains, which can grow at temperatures up to 41°C (Hubalek et al., 1998), and is especially surprising given the high body temperatures associated with TBRF [up to 41.7°C (107°F) in humans] (Dworkin et al., 1998; Talagrand-Reboul et al., 2018). Further studies are required, however, to assess if *in vitro* sensitivity to elevated culture temperature is unique to *B. turicatae* or if this is common among TBRF spirochetes. Next, to evaluate susceptibility of *btpA* mutants to oxidizing agents, a semi-solid media plating-based approach was utilized (Raffel et al., 2018; Bourret et al., 2019). Bourret et al. recently showed that TBRF spirochetes are significantly more resistant to H_2_O_2_ relative to *B. burgdorferi*, hypothesizing that BtpA could be involved in this differential susceptibility (Bourret et al., 2019). Interestingly though, we failed to identify a role for BtpA in resistance to oxidative stress produced by H_2_O_2_. The ability of *B. turicatae* to resist higher levels of H_2_O_2_ relative to *B. burgdorferi* appears to therefore be independent of BtpA, indicating the existence of other unidentified mechanisms found in TBRF *Borrelia*, but not in LD *Borrelia*, that are involved in resistance to ROS. In contrast, we observed a modest contribution of BtpA in the defense of *B. turicatae* against *t*-butyl peroxide. This difference is likely due to the fact that organic peroxides primarily target polyunsaturated fatty acids in *Borrelia* cell membranes (Boylan et al., 2008), while oxidative stress produced by H_2_O_2_ occurs after it diffuses across the cell envelope and presumably produces hydroxyl radicals (^∙^OH) as the result of Fenton chemistry (Imlay, 2003). Future studies will be required to determine whether BtpA protects the *B. turicatae* cell membrane against lipid peroxidation caused by organic peroxides, or whether it confers resistance to oxidative stress by a different mechanism.

A potential limitation of this study with respect to interpretation of the phenotype of *btpA* mutants upon treatment with *t*-butyl peroxide is the absence of genetic complementation. Complementation would ensure that this increased sensitivity can be directly attributed to the loss of *btpA* specifically, and not due to an off-target effect associated with either randomly acquired mutations or polar effects introduced via mutational strategies. However, as noted above, genetic manipulation of TBRF spirochetes is still in its infancy. As such, successful complementation of a targeted mutagenesis approach in TBRF spirochetes has only been reported in one study using *B. hermsii* (Raffel et al., 2014). Importantly, this study utilized a method of plasmid incompatibility to perform complementation of a gene on a small (28-kb) linear plasmid. This method requires integration of an antibiotic resistance marker into the plasmid of interest in the wild-type *Borrelia* strain. Genomic DNA, isolated from a bacterial clone containing the plasmid with the resistance marker, is transformed into the mutant strain in order to replace the mutated plasmid with a plasmid containing the wild-type copy of the gene. This method is not possible in the case of our study however, as *btpA* is encoded on the ~1 Mb chromosome. Other possible complementation methods, such as site-specific integration and use of a shuttle vector, are also problematic. First, a stable shuttle vector has not yet been described for use in *B. turicatae*. Second, *cis*-based complementation by integration and restoration of *btpA* at the original location in the genome is complicated by the location of the gene in the middle of an operon, as well as organization of the genes flanking the operon. Additionally, integration of the gene into another site in the *btpA* mutant genome is complicated by the lack of a commonly used integration site that has been empirically proven not to lead to polar effects. The region analogous to the *bb0445-bb0446* integration approach is not conserved in *B. turicatae* and contains a unique gene in this intergenic region (Li et al., 2007; Promnares et al., 2009; Yang et al., 2009; Zhang et al., 2009; Pitzer et al., 2011; Moon et al., 2016). While a green fluorescent protein allele has been integrated into the genome of *B. turicatae*, integration occurred at an unknown location, and thus could lead to other potential off-target effects (Krishnavajhala et al., 2017). The lack of complementation in this study highlights the need for further development of genetic tools for use in *B. turicatae*. Because restoration of *btpA* expression in the mutants will be essential for studies intended to delineate the role of *btpA* during tick colonization and transmission, we are currently developing an alternative site-specific integration approach that will be suitable for complementation in *B. turicatae*. To overcome the lack of complementation, our study used two independent clones generated with different mutational constructs, and similar phenotypes were seen with each mutant. The probability that these independently generated mutants have similar randomly acquired mutations is not likely. We did observe subtle changes in expression of *bt0791* in each of the *btpA* mutants, indicating polar effects may have been introduced by the mutational strategies used. However, *bt0791* expression was slightly increased in the *btpA*::P*flgB*-*aacC1* mutant, whereas expression was slightly decreased in the *btpA*::*aacC1* mutant. The observation that these mutants exhibited similar oxidative stress phenotypes, but contained subtle, but opposite, changes in *bt0791* expression indicates that polar mutations are likely not the reason for decreased resistance to *t-*butyl peroxide. Additionally, bacterial thymidine kinases are not known to be involved in resistance to oxidative stress, further arguing against the *t*-butyl peroxide phenotype being associated with polar effects. Finally, our results demonstrating decreased resistance to *t*-butyl peroxide agree with previous heterologous expression experiments (Guyard et al., 2006). Therefore, these findings support the supposition that the mutants' increased sensitivity to *t*-butyl peroxide is due to loss of *btpA* and not an off-target effect, despite lack of complementation.

We observed only modest defects in the ability of *btpA* mutants to withstand environmental stresses *in vitro*, but it was still possible that BtpA could be required for mammalian infection. However, using a murine model of RF, we found that BtpA was not required for the mammalian phase of the enzootic cycle. The modest defects in the *btpA* mutants *in vitro* and their lack of attenuation *in vivo* suggest that compensatory mechanisms exist that render BtpA dispensable in these assays. Interestingly, there is another HtrA family serine protease encoded in the genomes of TBRF spirochetes. BT0104, referred to hereafter as BtHtrA, is a chromosome-encoded protein that is conserved among TBRF and LD *Borrelia* (Hyde and Johnson, 1986; Fraser et al., 1997; Penningon et al., 1999; Guyard et al., 2006; Miller et al., 2013). BtHtrA and TBRF spirochete orthologs have not been investigated, but the *B. burgdorferi* homolog, BB0104/BbHtrA, has been extensively studied (Coleman et al., 2013, 2016, 2018; Gherardini, 2013; Kariu et al., 2013; Russell and Johnson, 2013; Russell et al., 2013; Ye et al., 2016). Several of the substrates recognized by BbHtrA have been identified as virulence factors of *B. burgdorferi*, including BB0323, P66, BmpD, and CheX, and BbHtrA appears to also play a key physiological role, as mutants demonstrate morphological and structural defects (Coleman et al., 2013; Kariu et al., 2013; Ye et al., 2016). In line with a role in physiology and processing of virulence factors, BbHtrA is required for mammalian infection of *B. burgdorferi* (Ye et al., 2016). Intriguingly, while the function of BbHtrA has not been investigated with respect to oxidative and nitrosative stresses, *bb0104* mutants do exhibit a growth defect at 37°C (Ye et al., 2016). These observations may indicate that BtHtrA could compensate for loss of *btpA* with respect to heat shock, mammalian infection, and possibly ROS/RNS resistance. However, two observations contradict this possibility. First, alignment of BtHtrA and BtpA revealed only 19.8% identity (Hyde and Johnson, 1986; Fraser et al., 1997; Penningon et al., 1999; Guyard et al., 2006; Miller et al., 2013). Similarly, BhHtrA and BhpA of *B. hermsii* share only 19.3% identity (Guyard et al., 2006). Furthermore, as mentioned by Guyard et al., BtpA homologs have a >100 bp C-terminal extension of unknown function not present in other HtrA family proteases (Guyard et al., 2006). Second, cellular localization experiments of BbHtrA of *B. burgdorferi* indicate surface localization (Russell and Johnson, 2013), as well as presence in the periplasm (Kariu et al., 2013). BhpA of *B. hermsii*, however, is only located intracellularly (Guyard et al., 2006). The lack of identity of BtHtrA and BtpA homologs and the difference in cellular localization indicates likely functional distinction between these two proteins.

While BtpA is dispensable for mammalian infection, it remains possible that BtpA has a function that is critical during another aspect of the TBRF spirochete enzootic cycle. Specifically, BtpA might be required for vector acquisition, vector colonization, or transmission from the vector to the mammal. Interestingly, Bourret et al. recently demonstrated that the salivary glands of *Ornithodoros turicata*, the tick vector for *B. turicatae*, were a highly oxidative environment (Bourret et al., 2019). Because TBRF spirochetes persistently colonize both the salivary glands and midgut of ticks, whereas LD spirochetes only persistently colonize the midgut, this may lead to an increased need for proteins involved in resistance to oxidative stress for TBRF spirochetes. We observed only a modest increase in the susceptibility of *btpA* mutant strains to *t*-butyl peroxide *in vitro*, but this assay does not fully recapitulate what TBRF spirochetes encounter in the tick environment. First, we treated the spirochetes with oxidative agents for 2 hours and then tested survival by plating. However, TBRF spirochetes have been noted to remain viable and infectious in unfed *Ornithodoros* ticks for several years (Barbour and Schwan, 2019). Therefore, if BtpA is required by *B. turicatae* to resist prolonged exposure to oxidative stress, the assay utilized may not detect this. Second, there are likely multiple assaults faced by TBRF spirochetes in the tick salivary glands that cannot be simulated by an *in vitro* oxidative stress assay. In addition to genes related directly to oxidative stress being transcribed in the *Ornithodoros* salivary glands (Bourret et al., 2019), Araujo et al. recently reported that several other anti-microbial genes are expressed in Argasid ticks, including microplusins, which can affect protein folding (Araujo et al., 2019). Perhaps the combined pressures of oxidative stress and other assaults renders BtpA essential for survival in the salivary glands. Finally, it is also possible that BtpA could be important for *B. turicatae* transmission from tick to mammal. It should be noted that the dose used for inoculation of mice in our murine infection experiments (10^2^ bacteria) is at least 10-fold higher than the number of spirochetes that can possibly be deposited into the skin during *Ornithodoros* tick feeding (Boyle et al., 2014). Therefore, it is conceivable that this higher dose could overcome a potential necessity for BtpA upon entry into the mammal, as more spirochetes would increase the chances of at least one bacterium surviving the initial infection event and associated mammalian immune defenses. Future studies will evaluate the capacity of *btpA* mutants to successfully complete the tick phase of the *O. turicata-B. turicatae* enzootic cycle.

In summary, we found that BtpA, as well as proteins encoded by adjacent chromosomal genes (BT0790 and BT0791), are produced in response to culture at mammalian body temperature, consistent with a role in mammalian infection and prior transcriptional studies. The genes encoding BT0790, BtpA, and Bt0791 were subsequently shown to be transcribed in an operon. To determine if BtpA was required for resistance to environmental stresses and during mammalian infection, we inactivated *btpA* in *B. turicatae* using two mutational strategies. *btpA* mutants showed no defect in response to heat shock. However, the mutants did exhibit a modest increase in sensitivity to the oxidative agent, *t*-butyl peroxide, suggesting a possible role for BtpA in resistance to oxidative stress. Finally, *btpA* mutants were fully infectious in a murine model of RF. Future studies will determine if BtpA is required for acquisition/colonization in the tick and subsequent transmission from tick to mammal.

## Data Availability

The datasets generated for this study are available on request to the corresponding author.

## Author Contributions

JB, CJ-L, TB, and JEL contributed to project conception, design of the study, or oversight of experiments. CJ-L, AZ, CR, and JIL performed experiments in the study. JB, CJ-L, and TB performed the data analysis and statistical tests and wrote the manuscript. All authors contributed to manuscript revision and read and approved the submitted version.

### Conflict of Interest Statement

The authors declare that the research was conducted in the absence of any commercial or financial relationships that could be construed as a potential conflict of interest.
